# Filling gaps in visual motion for target capture

**DOI:** 10.3389/fnint.2015.00013

**Published:** 2015-02-23

**Authors:** Gianfranco Bosco, Sergio Delle Monache, Silvio Gravano, Iole Indovina, Barbara La Scaleia, Vincenzo Maffei, Myrka Zago, Francesco Lacquaniti

**Affiliations:** ^1^Department of Systems Medicine, University of Rome “Tor Vergata”Rome, Italy; ^2^Centre of Space Bio-medicine, University of Rome “Tor Vergata”Rome, Italy; ^3^Laboratory of Neuromotor Physiology, IRCCS Santa Lucia FoundationRome, Italy

**Keywords:** visual motion extrapolation, eye movements, manual interception, internal representations, gravity

## Abstract

A remarkable challenge our brain must face constantly when interacting with the environment is represented by ambiguous and, at times, even missing sensory information. This is particularly compelling for visual information, being the main sensory system we rely upon to gather cues about the external world. It is not uncommon, for example, that objects catching our attention may disappear temporarily from view, occluded by visual obstacles in the foreground. Nevertheless, we are often able to keep our gaze on them throughout the occlusion or even catch them on the fly in the face of the transient lack of visual motion information. This implies that the brain can fill the gaps of missing sensory information by extrapolating the object motion through the occlusion. In recent years, much experimental evidence has been accumulated that both perceptual and motor processes exploit visual motion extrapolation mechanisms. Moreover, neurophysiological and neuroimaging studies have identified brain regions potentially involved in the predictive representation of the occluded target motion. Within this framework, ocular pursuit and manual interceptive behavior have proven to be useful experimental models for investigating visual extrapolation mechanisms. Studies in these fields have pointed out that visual motion extrapolation processes depend on manifold information related to short-term memory representations of the target motion before the occlusion, as well as to longer term representations derived from previous experience with the environment. We will review recent oculomotor and manual interception literature to provide up-to-date views on the neurophysiological underpinnings of visual motion extrapolation.

## Introduction

**Figure 1:**
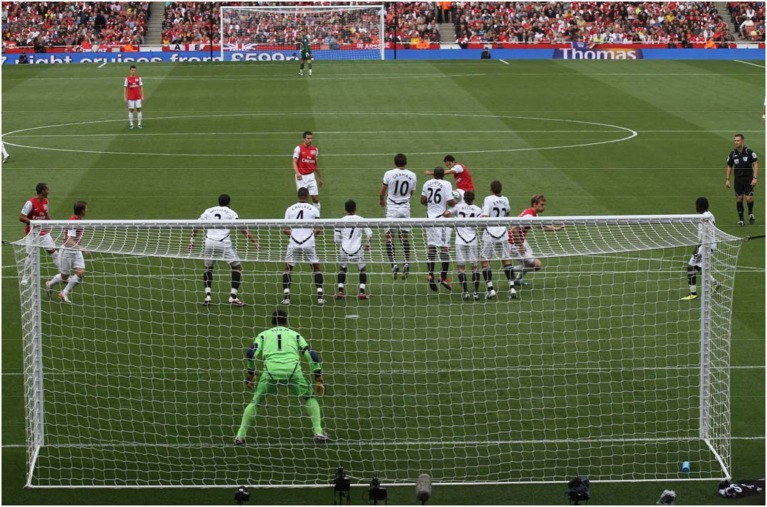
**Free kick play in a soccer game, seen from behind the goalkeeper**. In order to block the opponent’s shot, defendants stand side-by-side as a barrier, which partially occludes vision of the ball to the goalkeeper.

**Figure 2:**
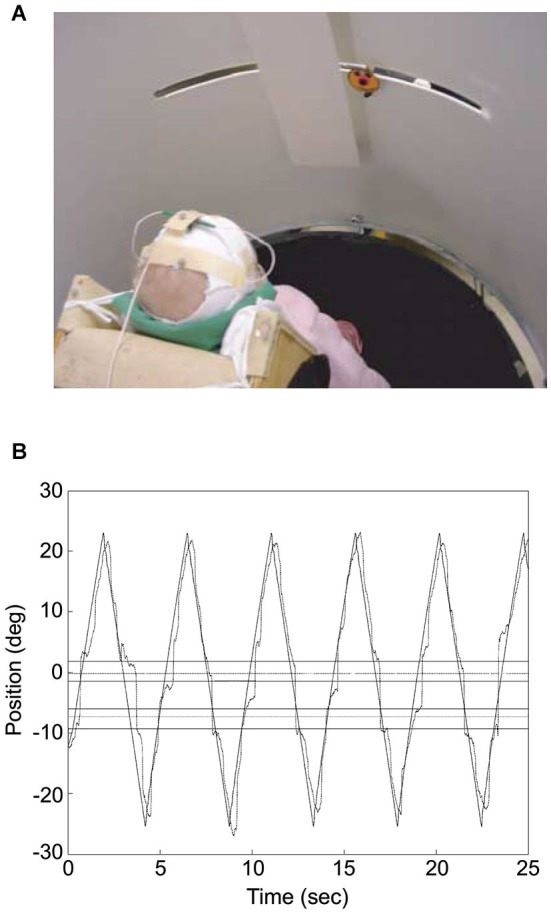
**Infant tracking a target moving through a visual occluder. (A)** The photograph illustrates the experimental set up. The infant was placed at the center of a cylinder, which screened him/her from external distractors. The “happy-face” object moved along a slit 60 cm long and disappeared transiently behind the rectangular screen. Eye movements were recorded by means of electro-oculography while infant’s head movements and object motion were acquired through a motion analysis system. **(B)** Infant’s gaze tracking plotted along with the object motion for an individual trial. Gaze direction was derived from the sum of head and eye directions, with head direction computed from the relative positions of three markers placed on the infant’s head with respect to the axis of rotation of the cylinder where the infant was placed (which was approximately aligned to the infant’s body axis). The outer horizontal lines represent the boundaries of the occluder, whereas the inner horizontal lines delimit the positions where the object was totally occluded. The horizontal lines in between the inner and outer ones represent the 2° tolerance outside which gaze was considered to have reached the other side of the occluder. The zero value along the ordinate corresponds to the middle position along the path of the target object. Modified with permission from von Hofsten et al. (2007).

A remarkable challenge our brain must face constantly when interacting with the environment is represented by ambiguous and, at times, even missing sensory information. This is particularly compelling for visual information, the main sensory system we rely upon to gather cues about the external world. The issue can be exemplified by typical situations occurring in the sports field. Let’s imagine a scene from a soccer game, such as that illustrated in Figure 1: a goalkeeper dives to save the goal from an opponent’s long distance shot, with the ball being hidden by other players that occlude the goalkeeper view. Despite the temporary disappearance of the ball from his field of view, the goalkeeper may be able to intercept the shot successfully and save the goal. Apparently, the goalkeeper has been able to predict where and when to intercept the shot by “guessing” the motion of the ball through the periods of absent visual motion information. In the wilderness, similar mechanisms may be important for survival. Predators, for example, are able to catch the escaping prey even though visual obstacles like trees and bushes may hide it from view, underlining the potential evolutionary pressure for the development of predictive mechanisms, which might compensate for the lack of sensory information. This ability appears to be acquired rather early during post-natal development in humans. Von Hofsten and colleagues have shown, in fact, that 4–5-months-old infants can anticipate where and when an occluded moving object will reappear (see Figure 2), and their predictions may reflect a representation of object velocity during the occlusion (Rosander and von Hofsten, 2000; von Hofsten et al., 2007).

Ample debate exists in the literature about the putative mechanisms that may account for the ability of predicting the objects’ visual motion through periods of transient disappearance. An experimental paradigm that has been commonly adopted to investigate this issue is the prediction motion (PM) task whereby subjects estimate the time of arrival of a hidden visual target at a specific location indicated by the experimenter. One early hypothesis to explain behavioral responses to PM tasks involved a clocking mechanism by which observers may initially estimate the time to contact (TTC) using on-line optical variables, and then, at the time of object’s disappearance, they may use a clocking process to count down time until the estimated TTC equals zero (Tresilian, 1995). While cognitive clocking mechanisms may account for PM responses to approaching objects along the line of sight (looming stimuli), they have been shown to generalize poorly to other behavioral situations involving, for example, lateral motion of the visual target (Lyon and Waag, 1995; DeLucia and Liddell, 1998). It has been, then, hypothesized that the brain adopts motion extrapolation mechanisms involving internal or cognitive representations of the object’s visible motion (Schiff and Oldak, 1990; Lyon and Waag, 1995). There is evidence that visual extrapolation can be sustained by a combination of several, perhaps interdependent, processes such as imagery, corollary eye movement signals, and attentional shifts. For example, observers may imagine the object continuing its motion after disappearance, and then respond as the imagined object gets to the target. Interestingly, a recent fMRI study has shown that visual motion imagery engages specific activations in occipital cortex, hMT/V5+ and IPL, regions involved in visual motion processing (Kaas et al., 2010). Oculomotor tracking of the invisible target may also reflect mental imagery processing, providing additional information to the representation of the occluded target trajectory (Huber and Krist, 2004). Moreover, motion imagery may be associated with visuospatial attention shifts (de’Sperati and Deubel, 2006). Therefore, visual extrapolation processes may also involve shifting an attentional “spotlight” (Lyon and Waag, 1995; Kerzel, 2003a,b). Further evidence that visual motion extrapolation may demand additional cortical processing and attentional resources comes from a series of experiments, which showed that absence of visual information may prolong the latency to suppress an anticipatory movement to intercept a moving target and affect the corticospinal excitability (Marinovic et al., 2010, 2011).

A fundamental question to understand the nature of the visual extrapolation processes concerns, however, the type of information potentially contributing to the internal representation of the hidden target motion built by the CNS. In this respect, much insight has been gained, over the last few decades, from studies examining the oculomotor and the manual interceptive behavior.

## Evidence from oculomotor studies

Experimental paradigms involving transient disappearance of the visual target have been used extensively to study the predictive component of pursuit and saccadic eye movements. For example, when visual feedback is transiently removed during ocular pursuit, it has been shown that extra-retinal input may continue to drive the smooth pursuit, albeit at a reduced gain (Mitrani and Dimitrov, 1978; Morris and Lisberger, 1987; Pola and Wyatt, 1997). Continuation of smooth pursuit in the absence of a visual target is believed to be under volitional control and mediated by the subject’s intention, as it is driven by the expectancy of the target to reappear (Becker and Fuchs, 1985; Bennett and Barnes, 2003; Madelain and Krauzlis, 2003; Barnes and Collins, 2008). In particular, a series of seminal studies carried out by Bennett, Barnes and collaborators shed some light on the variables the oculomotor control processes might take into account to maintain ocular tracking of hidden targets. By imposing a systematic change in the target velocity during the invisible portion of the trajectory, Bennett and Barnes (2004) were able to show that recovery in eye velocity after the loss of visual feedback was scaled and hence predictive of the upcoming target velocity. They proposed that predictive changes of eye velocity were the result of scaled modifications of an internal gain signal. In a later study (Bennett and Barnes, 2005), the same authors used target blanking of variable duration to examine whether the timing of this anticipatory recovery could be influenced by the duration of the target disappearance. They found that the recovery of the eye velocity was indeed timed to the target disappearance, perhaps as result of reactivation of variable gain mechanisms acting on the visuomotor signals, which drive ocular pursuit. Moreover, when subjects were required to pursue accelerating targets that underwent transient occlusions and were presented in either random or blocked order with respect to the acceleration conditions, they showed anticipatory smooth pursuit prior to target motion onset, scaled to the velocity generated by the target acceleration, at least during blocked presentations. This scaling in the eye velocity prior to the target reappearance led to the interpretation that the oculomotor control system might use a target velocity representation to drive predictive pursuit (Bennett and Barnes, 2006a).

Similarly, by using an occluded onset pursuit paradigm, Collins and Barnes (2006) found that smooth pursuit started progressively earlier with respect to the target appearance as the occlusion interval increased, and with increasingly higher pursuit gains by the time the target appeared. The prolongation of the anticipatory smooth pursuit throughout the occlusion was interpreted again as reflecting storage of velocity information in the form of working memory (Collins and Barnes, 2006).

Both pursuit and saccadic control use predictive information about target motion during extrapolation of occluded targets. In fact, subjects can correct for the error in eye position due to the decrease in pursuit gain, by producing saccades that place the eye ahead of the extrapolated position of the occluded object (Bennett and Barnes, 2006b). Further evidence for interaction between pursuit and saccadic system in the ocular tracking of occluded targets was provided by a study of Orban de Xivry et al. (2006), who found that eye velocity at target reappearance was influenced by expected target velocity, whereas saccades reflected the expected change of target position (see also de Brouwer et al., 2001).

Besides target velocity, also target acceleration information appears to be used by the oculomotor control system for the extrapolation of occluded targets. Bennett et al. (2007) adopted an experimental paradigm where target’s position and velocity during the occlusion and at reappearance could not be predicted without extracting the acceleration of the target before its disappearance. They found that smooth and saccadic eye movements discriminated between different levels of acceleration after an initial exposure of at least 500 ms (Bennett et al., 2007). Despite the fact that this result was obtained by using non-ecological experimental conditions, its significance may have some bearing also with respect to more natural situations where moving objects, because of the effects of external forces (such as gravity), do not move at constant velocity.

Visual extrapolation mechanisms driving eye movements can also take into account dynamic directional changes of the target motion, as shown by the results of experiments where subjects had to track targets along two-dimensional paths. For example, Mrotek and Soechting instructed subjects to track targets, which moved initially along a straight path and then followed the arc of a circle, just before disappearing behind an occluder (Mrotek and Soechting, 2007). When the target re-emerged after following the curvilinear path through the occlusion, gaze behavior indicated that subjects were able to predict the curvilinear target motion through the occlusion, compatible with the idea that extrapolation of occluded targets may include expectations about the time-varying behavior of the target motion.

Further evidence along these lines comes from a study, which investigated smooth pursuit and saccadic responses during occlusion of targets moving along circular paths (Orban de Xivry et al., 2008). In this study, subjects pursued visual targets moving along counterclockwise circular paths that could vary randomly across trials in both radius and frequency. The target, initially visible for at least a half-circle, underwent three successive periods of occlusion ranging from 0.4 to 1 s alternated with visible periods lasting between 0.8 and 1.5 s. Analysis of smooth pursuit responses showed that, during target occlusions, the smooth pursuit heading direction could predict accurately the heading of the target. This result was best explained by a dynamic memory model that included time-varying information about target velocity during the occlusion period, suggesting that predictive pursuit was driven by a time-varying internal representation of target motion. In addition, it was found that the predictive smooth pursuit system influenced the amplitude of the predictive saccades but not vice versa, underlining the complexity of the interactions between the pursuit and the saccadic system. Besides maintaining smooth pursuit in the absence of the visual stimulus, internal representations based on expectancy of predictable target motion can also drive smooth eye deceleration, direction reversal, and subsequent acceleration (Kveraga et al., 2001).

Dynamic internal representations driving eye movements do not incorporate only short-term visual information acquired before target’s disappearance, but they may also reflect cognitive factors and memory of target motion accumulated with previous experience. This idea was tested directly by a series of experiments, in which accelerative and decelerative target motions were presented in either random or blocked-order, and catch trials with different target acceleration were interleaved randomly between blocked-order trials. It was found that the recovery of the smooth pursuit following the target’s occlusion was better scaled in blocked-order than in random-order trials. Particularly, in blocked-order trials, the reduced smooth pursuit gain during occlusion was compensated by modulation of the saccade amplitude, such that the total eye displacement matched well the target displacement. In catch trials, experimental subjects could show scaled responses to the unexpected change in target acceleration, albeit they also exhibited transfer effects from the preceding blocked-order trial. Overall, these findings were interpreted as evidence that short-term predictions derived from online information can be combined with long-term memorized information from previous trials to generate accurate predictions of the occluded target trajectories (Bennett et al., 2010b). Similar predictive mechanisms, in effect, have been shown to account also for smooth pursuit initiation and oculomotor responses to sudden perturbations of the target motion (Kowler and Steinman, 1981; Kowler et al., 1984; Tabata et al., 2008).

By simulating naturalistic conditions, recent studies of oculomotor behavior have indicated that long-term information might include also experience-based models of physical interactions between the visual target and the environment. In a series of experiments, Diaz et al. created an immersive virtual racquetball environment, which allowed them to study both eye and hand interceptive movements made by unskilled players to hit virtual balls that were projected in a parabolic arc consistent with the effects of gravity and bounced on ground before arrival (Diaz et al., 2013a). The ball trajectories were manipulated parametrically such that balls varied in the launch point, the location of the bounce, and the location where they passed the observer. In addition, balls could differ in elasticity, and thus could follow different trajectories after the bounce even when they had the same pre-bounce trajectories. Consistent with the idea that predictions based on internal models of the physical properties of the environment may represent a significant component of oculomotor control, Diaz and colleagues found that pre-bounce saccadic movements accounted for changes due to both ball velocity and elasticity and predicted the time and location of the ball after the bounce. Moreover, subjects’ oculomotor behavior was not affected by a 100 ms period of visual occlusion of the ball’s trajectory immediately after the bounce, reinforcing the idea that memorized information about the dynamic properties of the moving object is incorporated in the oculomotor plan (Diaz et al., 2013b).

In the same vein, target motion congruent with the natural motion of an object rolling on the ground elicits faster anticipatory pursuit responses compared with incongruent motion, suggesting that information about natural object kinematics acquired through daily-life experience is taken into account by oculomotor control processes (Souto and Kerzel, 2013). In this respect, gravity represents a major factor since it affects invariably the motion of falling objects by imposing a constant accelerative force. While most of the evidence in favor of the idea that the brain has internalized information about gravity effects for predictive control of motor behavior comes from manual interceptive studies (which we will review in the next section), there is recent evidence that also the oculomotor control may exploit such a-priori knowledge. Delle Monache et al. (2015) analyzed the oculomotor behavior of head-fixed subjects. Subjects shifted a mouse cursor to intercept computer-simulated ballistic trajectories, which could be occluded for variable intervals before interception. The law of motion of the trajectories could be either congruent with Earth’s gravity (1 g) or perturbed with weightlessness (0 g) or hypergravity (2 g) effects. Analysis of the overall oculomotor behavior during the manual interception task and of isolated periods of smooth pursuit of the ball trajectories showed that eye movements depended significantly on the target law of motion and occlusion, in ways compatible with the view that predictive mechanisms based on implicit knowledge of gravity effects may contribute to oculomotor control (Delle Monache et al., 2015).

In sum, the idea emerging from reviewing these studies is that control of predictive eye movements to overcome missing visual information may take advantage of an internal representation of the target motion, which combines time-varying information (target velocity, acceleration) derived from vision of the target motion prior to its disappearance and prior knowledge of the target motion derived from heuristics and from internalization of invariant physical characteristics of the environment.

## Evidence from manual interception studies

Hand interceptive actions represent another particularly interesting experimental model to study visual extrapolation mechanisms. These actions require accurate estimation of the time and place for object interception, achieved through a fine interplay of feedback and anticipatory mechanisms to compensate for visuomotor delays, which, depending on the complexity of interceptive action, may make unusable the last 100–200 ms of visual information about the target motion (see Regan and Gray, 2000; Zago et al., 2008, 2009; Merchant et al., 2009 for reviews on the psychophysics and neurophysiology of interception). Thus, spatial and temporal aspects of the interceptive action might reflect predictions of the objects’ motion, especially when transient occlusions of the interceptive target or degradation of the visual motion information, such as apparent motion stimuli, are introduced within the experimental paradigm.

### Interception of occluded targets

One main issue that has been investigated by using visual occlusion paradigms relates, for example, to the early work of Whiting and collaborators who varied systematically onset and duration of visual occlusions of parabolic ball trajectories to determine potentially critical viewing time windows for successful interception (Whiting et al., 1970; Sharp and Whiting, 1974). These original studies have been replicated recently by using up-do-date technologies and it was found that the interceptive performance deteriorates dramatically only when vision is occluded from 150 to 200 ms prior to the movement onset, albeit the probability of successful interception may vary, to some extent, also with the overall length of time that the target is made visible (Marinovic et al., 2009; López-Moliner et al., 2010). For batting movements, however, precise timing of the interceptive actions may depend on whether visual information is available during the swinging movement (Brenner et al., 2014). In effect, there is also evidence that sudden changes in target direction could be estimated and the motion of the target extrapolated accurately with short viewing times comprised between 50–150 ms, suggesting that, under certain experimental conditions, even relatively small viewing times may allow accurate interception (Mrotek et al., 2004). Besides the extent of the visible motion, timing accuracy in motion extrapolation tasks has also been found to depend on the spatial properties of the target, such as its size. Target size effects seem to be strongly modulated by the target speed, according to the velocity transposition principle by which larger targets are perceived to be slower (Sokolov et al., 1997; Sokolov and Pavlova, 2003).

Target velocity, in fact, appears to be a major parameter of object motion taken into account by visual extrapolation processes for the timing of interceptive actions. Experiments in which a target moved across a computer screen with different velocity profiles and could be occluded either early or late in the trajectory, showed that subjects are able to use target velocity information acquired during the early part of visible trajectory to build a velocity representation of the occluded portion of the target motion and control the timing of the interceptive action (Dubrowski et al., 2000). The idea that internal representations of the target motion are built during the visible portion of the target motion and then used to update or predict the future trajectory is supported also by experiments that examined the effects on the temporal accuracy and the kinematics of interceptive movements of visual occlusions occurring after a sudden target velocity change. Compatible with this view, reduction of visual exposure affected movement variability, but not the velocity of the arm or the directional trend of timing errors (Teixeira et al., 2006).

Use of the velocity information available before target occlusion for guiding the interceptive action may be somewhat reminiscent of the control mechanisms underlying ocular pursuit of occluded targets described in the previous section. In effect, some analogies with the smooth pursuit system have emerged from a study, which examined the effects of occluding different portions of ball trajectories approaching from different directions on the lateral hand movements to catch the balls (Dessing et al., 2009). When the late portion of the ball trajectories was occluded, interceptive movements showed a significant spatial bias, which could be interpreted as a result of incomplete motion extrapolation. In particular, the authors hypothesized a reduction of the velocity gain similar to that observed in the smooth pursuit system (Dessing et al., 2009). Conversely, another study, which tried to disentangle the use of position and velocity information for the control of the interception position, suggested that target velocity might not contribute directly to the spatial estimates of the interceptive action (Brouwer et al., 2002). In this study, subjects hit visual targets moving at constant speed and disappearing after variable time intervals within either a stationary or a moving background. Hitting positions after the target disappearance denoted the use of velocity information only indirectly, perhaps via a speed-dependent misperception of position, implying also differential control of spatial and temporal estimates of the interceptive action (Brouwer et al., 2002).

Moreover, studies that involved more complex target motion patterns have indicated that visual extrapolation might include additional parameters besides target speed. For example, analysis of the initial direction of interception movements to hidden circular and oval trajectories pointed out that extrapolation of target motion was based on target position, velocity and curvature (Soechting and Flanders, 2008). Along the same lines, when subjects intercepted targets whose speed could be governed by three different laws of motion (i.e., constant speed, a power law relation between speed and curvature, or a sum of sinusoids), the initial direction of the interceptive movement could be predicted by assuming that target position was extrapolated using a combination of target velocity and distance from the finger to the target (Soechting et al., 2009). The studies mentioned thus far dealt mostly with motion of the interceptive target along the fronto-parallel plane. Conversely, for target motion approaching the observer (i.e., looming stimuli), classical views based on Gibson’s ecological approach have argued that TTC information can be derived directly from optical variables, like tau, that is, the ratio between the size of the object’s image on the retina and its expansion rate (Lee and Reddish, 1981). As mentioned in a previous section, when an occluder interrupts the flow of optical information, TTC would be counted down by relying on internal clocks (Tresilian, 1995). However, this view has been challenged by DeLucia (2004), who used computer-generated objects that approached the observer and were partially concealed by either stationary or moving occluders of variable size and shape. Results indicated that both types of occluders affected TTC judgments, suggesting that TTC estimates of partially occluded approaching objects is not based on tau but may involve information about heuristics and invariants.

In this respect, a growing body of research over the past decade has explored the possibility that, in addition to short-term memory of the target motion parameters, visual extrapolation for the control of interceptive actions may take into account both short and long-term representations derived from past history, cognitive cues or from internalized knowledge of the physical characteristics of the environment.

It has been shown, for example, that in PM tasks the target velocity of previous trials can affect current velocity estimates, suggesting that subsequently encoded velocity representations can be partially blended (Makin et al., 2008). Long-term representations of the target motion could be derived also from abstract categorical information, as indicated by a later study from the same research group, reporting that performance in a PM task was influenced by the color of the target, which was associated with different distributions of target velocities (Makin et al., 2009a).

**Figure 3:**
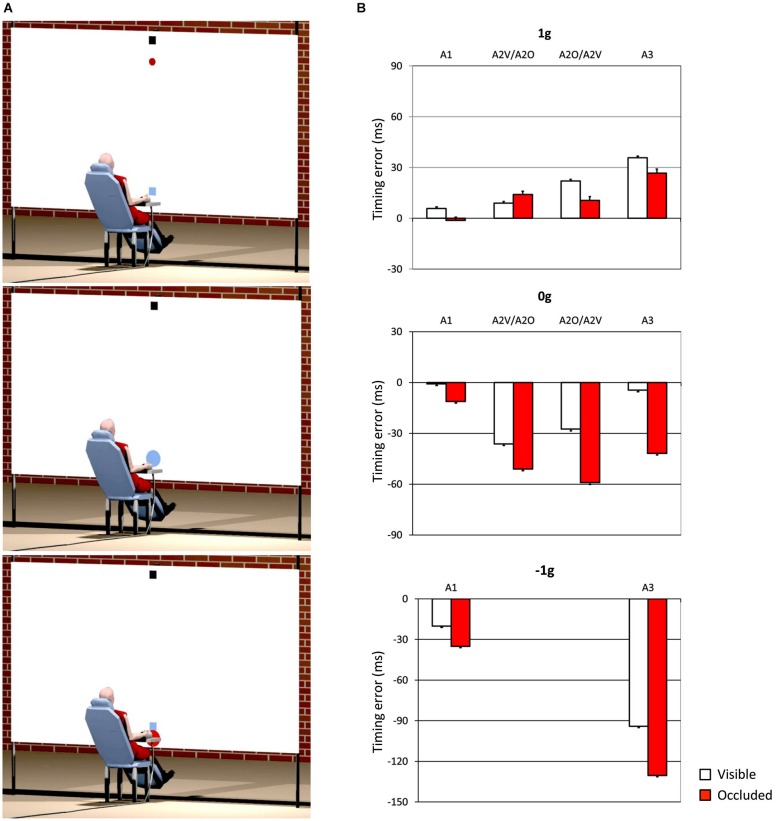
**Manual interception of transiently occluded targets moving along vertical trajectories**. **(A)** Illustration of the experimental setup. A red circular target moved downward on the screen from a start black box on the top (top panel). In each trial target motion could be either fully visible or transiently occluded. In occluded trials, the target was visible only during the first 300 ms and then disappeared from view. In separate trials, targets could be accelerated under gravity (1 g trials), decelerated under reversed gravity (−1 g), or move at constant speed (0 g). Subjects clicked a mouse button when they thought the target arrived at destination (the blue square). If subjects clicked within ±30 ms of target arrival, the target exploded blue to indicate successful interception (middle panel). If they intercepted too early (not shown) or too late (bottom panel), the target was flashed red at the incorrect position of interception. **(B)** Interception timing in the different experimental protocols tested by Zago et al. (2010). Experimental protocols could include the presentation, within each session, of one, two or three target acceleration types (A1, A2 and A3, respectively) and of either fully visible (V) or occluded (O) motion. Global mean values (±SEM) of time-to-contact (TTC) over all subjects of each group and all target motion durations are plotted for the indicated protocols. Positive (negative) TTC correspond to late (early) responses relative to target arrival at destination. Note the overall better timing performance for 1 g targets during occluded trials (red bars), in agreement with the use of predictive estimates based on anticipation of gravity effects.

One interesting aspect, which we also mentioned in the previous section, concerns the idea that internal models of physical characteristics of the visual environment can be built over experience or extensive practice and used to predict and intercept the motion of an object. This may be crucial to overcome limitations inherent to the visual system, like its poor sensitivity for accelerating motion despite the fact that in nature objects’ motion is often accelerated by gravity or decelerated by frictional forces (Werkhoven et al., 1992; Watamaniuk and Heinen, 2003). For interceptive actions of free-falling objects performed under full visual guidance, there is plenty of experimental evidence that TTC estimates reflect a combination of visual motion cues with prior knowledge of gravity effects on the object’s motion (see Zago and Lacquaniti, 2005; Zago et al., 2008, 2009; Lacquaniti et al., 2013 for reviews). The extent to which vertical accelerations could be extrapolated through brief occlusions was investigated by experiments that presented virtual targets descending on a blank screen accelerated by gravity, decelerated by reversed gravity, or at constant speed (Zago et al., 2010). Targets could disappear for a brief, variable period prior to arrival or could remain visible throughout their trajectory (see Figure 3A). Subjects’ response timing, during occluded trials, denoted better interceptive performance with 1 g targets compared to 0 g and −1 g targets (Figure 3B), arguing in favor of the idea that prediction of occluded vertical motion incorporates expectation of gravity effects (Zago et al., 2010).

Evidence in support of this view emerged also from a study, which evaluated the anticipatory muscle tensing (AMT) responses recorded from the biceps and flexor carpi radialis during catching of free-falling objects. Subjects were allowed full vision of the falling object in some trials, while in other trials they were instructed to keep their eyes closed. Moreover, in randomly selected trials the object stopped suddenly 12 cm above the hand, so to dissociate the effect of impact anticipation from the reflexive tactile response associated with the impact and determine the time scale of AMT control. The study found that the time-lags of the AMT responses were sufficiently long (121 ms, on average) to include acceleration in the anticipation of the falling object motion (Vishton et al., 2010). Predictive control based on acceleration timing information has been reported also with respect to the initiation of batting movements when subjects hit balls dropped from different heights and partially concealed, even though prospective control mechanisms could be involved in guiding the bat to ball interception (Katsumata and Russell, 2012).

The finding that movement initiation when intercepting free falling objects takes into account gravity acceleration information can be also seen within the probabilistic framework defined by the work of Faisal and Wolpert (2009). In this study, subjects had either to estimate the landing location of parabolic trajectories or intercept the trajectory at a pre-defined point by moving a virtual paddle on the PC screen. In separate experiments, either sensory or motor aspects of the task were manipulated by varying the portion of visible trajectory and the time allowed to move the virtual paddle, respectively. It was reported that subjects’ decisions when to start moving were statistically near optimal, provided individual sensory and motor uncertainties. In this respect, a-priori information about gravity acceleration could be seen as a factor reducing the sensory variability.

**Figure 4:**
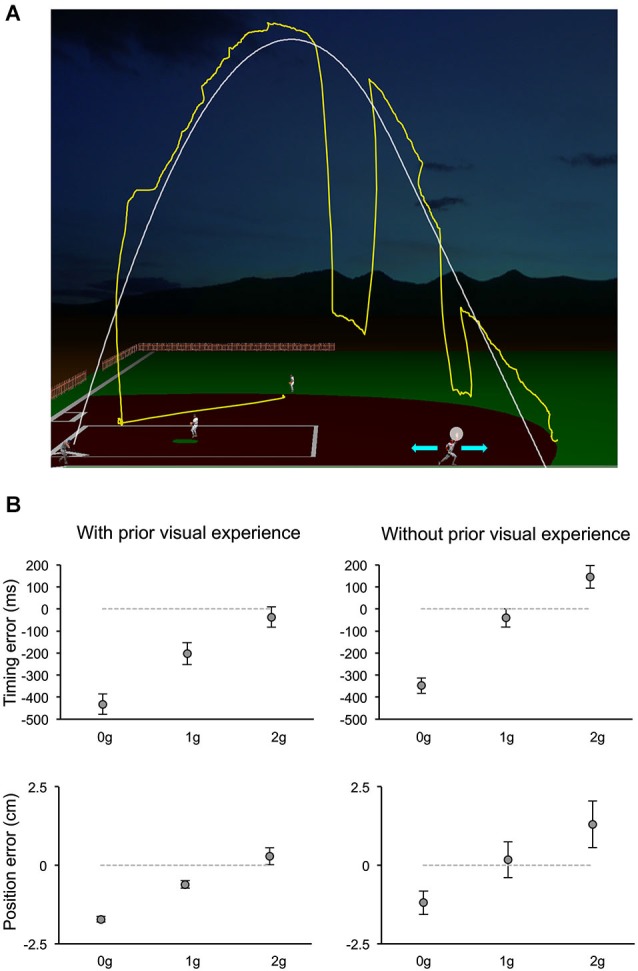
**Manual interception of occluded projectile trajectories**. **(A)** The visual scene simulated the baseball fly-ball play. The ball followed a parabolic path (white thin line), starting from the batter on the left bottom and landing on the right bottom. In separate sessions, ball trajectories could be either fully visible or occluded for variable time intervals before ball landing. In different trials, the natural ball motion could be either perturbed during the descending trajectory with effects of weightlessness (0 g) or increased gravity (2 g), or unperturbed (1 g). During each trial, participants kept the head fixed on a chin rest, while moving their eyes freely (the yellow trace shows a typical eye movement pattern). They intercepted the ball by controlling the outfielder’s movements with a PC mouse (cyan arrows refer to the possible movement directions) and indicated the interceptive event with a button press. The white semitransparent circle around the hand of the outfielder delimited the valid area for target interception. **(B)** Distributions of timing (TE, top panels) and positional errors (PE, bottom panels) recorded during the Occluded session of one experiment of Bosco et al. (2012). Data-points represent average values (±SEM) computed for each ball acceleration among subjects that either had full visual experience of the ball trajectories by performing first the Visible session (left column) or did not (right column). Positive and negative TEs denote late and early responses, respectively. Similarly, negative PEs indicate horizontal underestimation of the landing position of the ball, while positive values indicated overestimation. Consistent with the use of a-priori knowledge of gravity, subjects who performed first the Occluded session showed 1 g responses closest to correct, 0 g responses anticipated and spatially underestimated, and 2 g responses late and overestimated. Instead, subjects who had prior visual exposure with the perturbed trajectories, showed early and spatially underestimated responses to 0 g trials, but rather close responses to accelerated 1 g and 2 g trials, suggesting that knowledge of the visual properties of the perturbed trajectories was combined with predictive information about gravity effects. Modified with permission from Bosco et al. (2012).

The relative contribution of various information sources to the visual motion extrapolation processes underlying the control of the interceptive action has been examined more extensively by a recent study, which manipulated a computer-generated visual environment representing a baseball game (Bosco et al., 2012). Subjects intercepted simulated fly-ball trajectories, which were either fully visible or occluded for variable time intervals before ball landing (Figure 4A). The natural ball motion could be randomly perturbed during the descending limb of the parabolic trajectory with effects of either weightlessness or increased gravity, or it could remain unaltered. In addition, to examine the contribution of previous visual experience with the perturbed trajectories to the interception of the occluded targets, the order of visible and occluded sessions was permuted among subjects. Interception of fully visible targets denoted the combination of servo-controlled and predictive processes, whereas when intercepting occluded targets subjects relied mostly on predictive mechanisms. Ball motion predictions were based, however, on different information depending on previous visual exposure with the perturbed trajectories (see Figure 4B). Subjects without prior experience of the perturbed trajectories showed interceptive errors consistent with predictive processes based on a-priori knowledge of gravity. Instead, subjects who had been exposed to the fully visible trajectories showed interceptive responses to the hidden targets compatible with the idea that implicit knowledge of the perturbed motion was also taken into account for the extrapolation of occluded trajectories. This latter result is in agreement with another recent finding, which indicated that subjects can learn, after relatively short practice, to extrapolate and intercept, through periods of visual occlusion, complex targets’ motion resulting from arbitrary accelerations (de Rugy et al., 2012). Thus, at least within the context of the experimental conditions used by these two studies, internal models of arbitrary accelerations can be developed rapidly to afford visual extrapolation and interception of targets in absence of visual feedback. Furthermore, the evidence that a-priori knowledge of gravity is a primary factor taken into account for the interception of occluded projectile trajectories goes along with results of a perceptual study, which investigated the possible representational analogue of the projectile trajectory. In a series of experiments, subjects observed visual targets moving horizontally, either in the leftward or rightward direction, and vanishing after a variable distance along the motion trajectory. Subjects were asked to indicate the position of the vanishing target after a variable retention delay. Moreover, to control for oculomotor factors, in different experimental trials subjects were either constrained to ocular fixation or they could move their eyes freely. It was found that the horizontal and vertical components of the positional displacement in the subjects’ responses followed a different trend with respect to the length of the retention interval and to the oculomotor behavior. The perceptual localization of the vanishing position was shifted forward in the horizontal direction of motion with retention delays up to 150 ms and remained constant or decreased at longer delays. However, the degree of the horizontal displacement depended significantly on whether subjects held fixation or pursuit the visual target. Conversely, the vertical displacement of the perceptual localization was very small and constant for retention delays up to 150 ms, whereas it increased downward at constant rate with longer retention intervals, irrespective of the constrained or unconstrained ocular behavior, perhaps reflecting internalization of gravity information (De Sá Teixeira et al., 2013).

**Figure 5:**
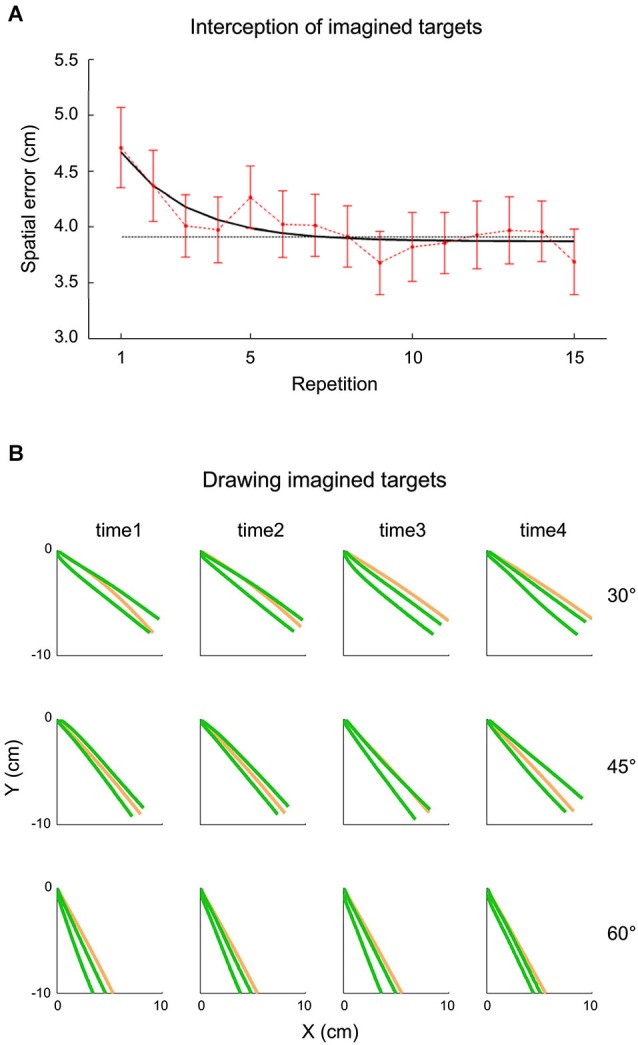
**Interception and drawing of imagined target motion**. A ball rolled down an incline with different kinematics depending on starting position and slope of the incline. Participants had to imagine that the ball kept moving after it was halted at the end of the incline and either intercept it or draw the ball trajectory as if it kept moving. **(A)** Effect of practice on spatial interceptive errors. Mean spatial error (±95% confidence interval, over all subjects and kinematic conditions) is plotted as a function of repetition (red). The black line represents the exponential best-fit, while the dotted gray line indicates the 95% decrement. **(B)** Panels show data recorded from a representative subject during the drawing task. The green lines represent the frontal plane projection of 95% confidence limits of hand paths over all repetition, while the orange lines indicate the actual ball paths. The relatively small spatial errors in the two tasks suggest that subjects were able to extrapolate to a good extent the motion of ball falling off the incline for both perceptual and visuomotor responses. Modified with permission from La Scaleia et al. (2014).

The idea that extrapolation processes underlying both perceptual and visuomotor responses might have access to an internal representation of gravity has been tested also within the context of inclined plane motion by a recent study carried out by La Scaleia et al. (2014). In these experiments, a ball rolled down an incline with different kinematics depending on the starting position and the slope of the incline. In different experiments, participants intercepted the ball as it fell off the incline, or they had to imagine that the ball kept moving after it was stopped at the end of the incline and either intercept it or draw with their hand in air the imagined ball trajectory. Although the most accurate behavioral performance was found with fully visible ball motion and haptic feedback of the hand-ball impact, also when participants intercepted or drew the imaginary trajectory, global aspects of the target motion, such as its path, speed and arrival time could be estimated well (see Figure 5). These results might imply that the paths and kinematics of balls rolling down inclined planes were extrapolated using both visual information and internal representations of the target motion (La Scaleia et al., 2014).

**Figure 6:**
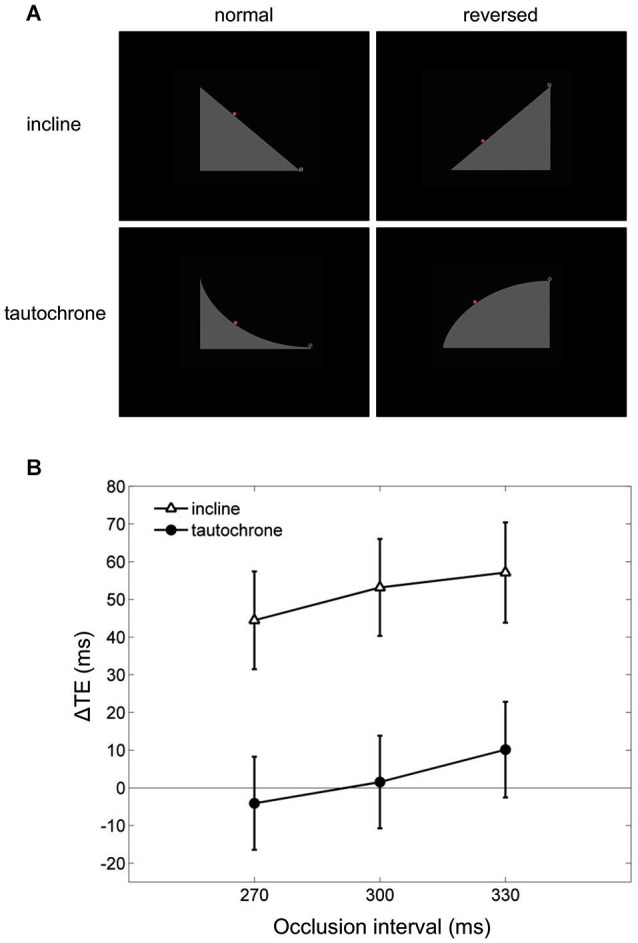
**Manual interception of familiar and unfamiliar trajectories**. **(A)** Visual scenes displayed during the experiments of Mijatović et al. (2014). Top and bottom rows show the inclined planes (familiar situation) and the tautochrones (unfamiliar situation). Left and right column panels depict the scenes used to produce “normal” and “reversed” kinematic conditions, which were delivered in separate sessions. The red dot represents the visual target rolling on either the inclines or the tautochrones, and the cross-hair at the right-most end of the path is the interception point. **(B)** Mean differences in timing error TE between the “reversed” and the “normal” sessions (ΔTE) are plotted as a function of occlusion duration. For each occlusion duration, ΔTE values were averaged across all motion durations, starting positions and subjects. Vertical bars around the means indicate 95% confidence intervals. Note that systematic differences in TE between “reversed” and “normal” kinematics, which may be indicative of a-priori assumptions of natural gravity, are found only for the familiar condition of the inclined plane (open triangles) but not for the tautochrone (filled circles).

Finally, visual extrapolation processes could be influenced by the familiarity with specific environmental conditions, which facilitates the interpretation of the underlying physical events. For example, time-to-contact estimates of approaching objects may be affected greatly by identity/familiarity information about the object, in addition to the retinal image expansion rates (Hosking and Crassini, 2010). Experiments in which participants intercepted targets sliding down either an inclined plane or a tautochrone (i.e., the cycloid curve along which a point mass slides to the bottom under gravity in the same time regardless of the starting position) examined this possibility more in depth (Mijatović et al., 2014). Although gravity acceleration affected the targets similarly in both cases, the inclined plane represented a familiar situation, whereas the tautochrone did not (Figure 6A). In these experiments, the gravity field could be congruent with either natural gravity or reversed gravity and the target motion could be occluded from view for variable time intervals before interception. Time shifts consistent with a-priori assumptions of natural gravity between the interceptive responses to natural and non-natural targets were found only for the familiar condition of the inclined plane but not for the tautochrone (see Figure 6B), suggesting that target motion extrapolation depends on integration of high-level cues about trajectory familiarity with lower-level target kinematics information (Mijatović et al., 2014).

### Interception of targets in apparent motion

In addition to the target occlusion paradigms we considered so far, there are other cases in which the kinematic information about a target displacement is incomplete or degraded. One such condition is represented by a target that suddenly disappears and reappears at a different position, so called “target jump”. The mechanisms involved in the interception of a jumping target will not be covered here, because they have been reviewed extensively elsewhere (Battaglia-Mayer et al., 2014). In this section, instead, we consider the case of intercepting long-range apparent motion (LAM). LAM is typically generated by flashing a stationary target in sequence at different locations along the path, with a wide spatial and temporal separation between successive locations. There is still no consensus as to whether LAM represents a high-level motion process distinct from low-level real motion (RM) or whether both RM and LAM result from the activation of spatio-temporal correlators by appropriate spatial and temporal combinations of the stimuli (see for instance, Adelson and Bergen, 1985; Watson and Ahumada, 1985; Cavanagh, 1992; Lu and Sperling, 2001). Irrespective of the specific mechanism involved, the spatio-temporal properties of LAM differ widely from those of RM (Gregory and Harris, 1984). Therefore, it is of interest to compare the interception mechanisms involved with LAM and RM.

Georgopoulos and colleagues studied the manual interception of targets moving at constant speed along a circle in RM or LAM in man and monkey (for a review, see Merchant and Georgopoulos, 2006). Subjects shifted a cursor to catch the target within a given interception zone. The spatial angular error increased linearly with target speed and was larger for LAM than RM (Port et al., 1996; Merchant et al., 2003). There was a similar trend of timing errors for both RM and LAM: subjects tended to be early with slowly moving targets, and late with fast moving targets. Overall, the results showed that there are similarities in performance between RM and LAM, but the performance is somewhat degraded in LAM.

**Figure 7:**
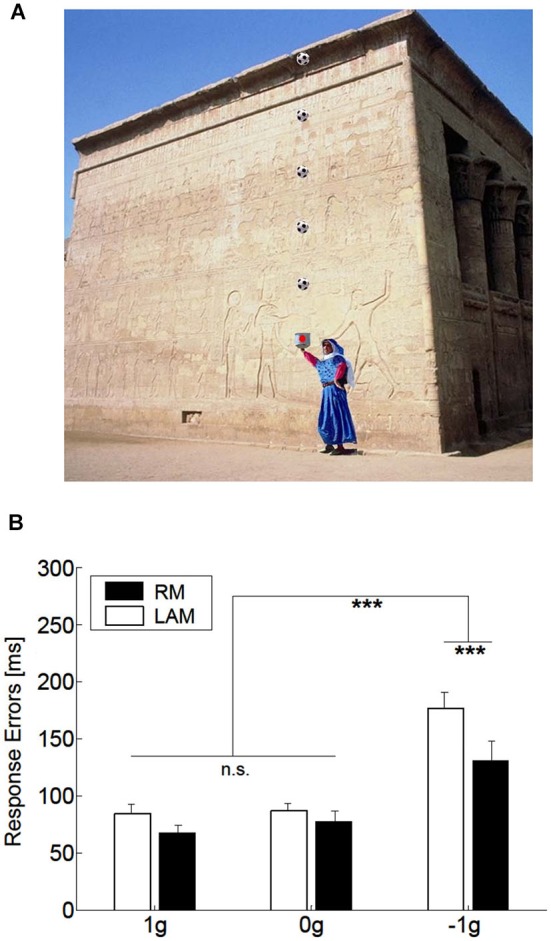
**Manual interception of targets in apparent motion**. **(A)** Visual scene and Long Range Apparent Motion (LAM) stimuli used in the experiments by Maffei et al. (2010). The soccer ball moved vertically relative to the scene first ascending from the box, bouncing on the building cornice, and then descending back into the box. For 1 g motion, ball acceleration was consistent with the effects of natural gravity, making the ball decelerating upward and accelerating downward. For −1 g motion, instead, ball acceleration was reversed relative to natural gravity and the ball accelerated upward and decelerated downward. For 0 g trials, the ball moved at constant speed. In Real Motion (RM) trials, the moving sphere was displayed every 16.7 ms, i.e., 60 Hz refresh rate. In LAM trials, the stationary ball was flashed for 50 ms at the 5 positions indicated in the picture, with temporal sequences congruent with 1 g, −1 g or 0 g law of motion. **(B)** The plot illustrates the effect of the law of motion (1 g, 0 g, −1 g) and of the type of motion (real, RM or Long Range Apparent Motion, LAM) on the mean absolute interceptive errors (±SEM). Black and white bars refer to RM and LAM, respectively. Asterisks denote statistically significant differences at *p* < 0.01. LAM visual stimuli produced a pattern of absolute interceptive errors similar to that observed for RM. Modified with permission from Maffei et al. (2010).

Manual interception of accelerating/decelerating LAM was studied by Maffei et al. (2010). Here, a target moved up and down at 1 g, 0 g, or −1 g and randomized initial speeds, and subjects had to press a button to intercept the sphere at the expected time of arrival at destination (Figure 7A). Because the target was never flashed at the arrival point in LAM, subjects had to extrapolate the stimulus traversing the vacant space in order to intercept the target. The resulting pattern of absolute timing errors was very similar for LAM and RM, with significantly smaller errors for 1 g and 0 g targets than −1 g targets (Figure 7B).

Overall, the studies reviewed here on the interception of occluded and apparent motion targets, have contributed to identify critical information the CNS may rely upon when filling the gaps in either degraded or missing visual information. Interestingly, some of the evidence presented in this section appears congruent with that obtained by analysis of the oculomotor behavior under similar experimental conditions, in that short-term low-level information about target kinematics may be combined with long-term cognitive associations derived from either external cues or familiarity conditions and internalized information about physical invariants. In particular, interceptive actions requiring haptic interactions with the visual target may make more compelling the use of a-priori knowledge about invariant physical forces acting on the object, such as the gravity acceleration.

## Do common predictive signals drive oculomotor and interceptive behavior?

As just noted above, strong commonalities with respect to the type of information contributing to the visual motion extrapolation processes emerged by examining the oculomotor and the manual interception literature (see Table 1 for a summary). Although these commonalities might imply that interceptive and oculomotor control share interactively predictive signals about the target motion, the issue is still controversial. On the one hand, psychophysical studies have raised the possibility that predictive information could be weighted differentially, or could even be entirely different (Wexler and Klam, 2001; Eggert et al., 2005). Moreover, based on analyses of the optical variables accounting for oculomotor and interceptive responses in a motion prediction task, Benguigui and Bennett (2010) argued for no functional relationship and independent control of ocular pursuit and interceptive timing. In later experiments, the same authors analyzed oculomotor and interceptive responses in a PM task using accelerating objects and found a general bias in the estimation of the reappearance position of the occluded objects that could be only in part explained by the oculomotor behavior during the occlusion. This led the authors to conclude that eye movements during occlusion may contribute, but do not uniquely specify information for accurate spatial or temporal estimation in the manual task (Bennett and Benguigui, 2013).

**Table 1:** **Summary table of the main factors contributing to the visual motion extrapolation processes, as reported by the oculomotor and manual interception studies reviewed in the article**.

	Eye movements	Manual interceptions
Target velocity	Bennett and Barnes (2004, 2005, 2006a,b), Collins and Barnes (2006), Orban de Xivry et al. (2006, 2008), von Hofsten et al. (2007), Mrotek and Soechting (2007)	Port et al. (1996), Dubrowski et al. (2000), Merchant et al. (2003), Teixeira et al. (2006), Soechting and Flanders (2008), Soechting et al. (2009)
Target acceleration	Bennett et al. (2007)	de Rugy et al. (2012), Katsumata and Russell (2012)
Target distance	de Brouwer et al. (2001), Orban de Xivry et al. (2006)	Port et al. (1996), Brouwer et al. (2002), Merchant et al. (2003), Soechting and Flanders (2008), Soechting et al. (2009)
Oculomotor efference copy	Mitrani and Dimitrov (1978), Becker and Fuchs (1985), Morris and Lisberger (1987), Pola and Wyatt (1997), Bennett and Barnes (2003), Madelain and Krauzlis (2003), Barnes and Collins (2008)	Bennett et al. (2010a), Makin and Poliakoff (2011); Delle Monache et al. (2015)
Target motion on earlier trials	Kowler and Steinman (1981), Kowler et al. (1984), Tabata et al. (2008), Bennett et al. (2010b)	Makin et al. (2008)
A-priori information about physical properties (i.e., elasticity, gravity)	Diaz et al. (2013a,b), Delle Monache et al. (2015), Souto and Kerzel (2013)	Maffei et al. (2010), Vishton et al. (2010), Zago et al. (2010), Bosco et al. (2012), Katsumata and Russell (2012), La Scaleia et al. (2014), Mijatović et al. (2014)
Cognitive associations with external cues	Not tested	Makin et al. (2009a)
Trajectory familiarity/ expectancy	Kveraga et al. (2001), Mrotek and Soechting (2007)	Mijatović et al. (2014)

On the other hand, there is also evidence that, even though independent control of arm and ocular responses may take place, concurrent arm movements can improve the gain of the smooth ocular pursuit during transient occlusions (Bennett et al., 2012). Analogously, the metrics of saccadic responses to memorized targets can be influenced significantly by the metrics of accompanying arm movements to the same targets, suggesting that signals related to the metrics of limb movements influence, at a programming stage, those controlling saccade metrics (Kattoulas et al., 2008). Further evidence in favor of shared predictive signals between oculomotor and manual interceptive control comes from a study which examined manual interceptive responses to occluded visual targets while subjects either fixated a designated point of the visual scene or tracked the visual target continuously (Bennett et al., 2010a). The study showed that eye movements could facilitate accurate TTC estimation in the interception task, indicating that pursuit of the visible portion of the object motion could be crucial in providing both extra-retinal and retinal signals to the predictive processes for the control of the interceptive timing (Bennett et al., 2010a). The importance of extra-retinal signals derived from eye movements for visual motion prediction has been remarked also by experiments testing the perceptual judgments made by subjects on whether a disappearing visual target would hit or miss a stationary goal. Results indicated again that motion direction predictions were significantly better when subjects pursued the target than when they maintained fixation (Spering et al., 2011).

Analogous differences were reported by Makin and Poliakoff (2011), who tested subjects with both a perceptual (a two-alternative forced choice task with respect to the time of target reappearance after the occlusion) and a PM task (subjects indicated with a button press the time of target reappearance after the occlusion). The two tasks could be performed under conditions that either enforced ocular fixation or allowed tracking the visual target. Subjects showed significantly different performance in both tasks under fixation compared to free eye movement conditions. More interestingly, eye movements during the occlusion were related to the subjects’ responses, suggesting that overlapping systems govern eye movements and perceptual/motor judgments on visual motion extrapolation tasks (Makin and Poliakoff, 2011). Along these lines, a recent study investigated the relationships between oculomotor behavior and interceptive performance while subjects intercepted, by acting on a computer mouse, projectile-like trajectories that could be either fully visible or occluded for variable time intervals before the interceptive event (Delle Monache et al., 2015). Interceptive errors were related by means of multivariate regression models to a number of oculomotor variables indicating where subjects directed their gaze throughout the trial, how many gaze shifts occurred during the trial, how stable was the oculomotor behavior close to the interception event, and how accurately subjects tracked either the target trajectory or the mouse cursor just prior to the interceptive response. Interestingly, when subjects intercepted the occluded targets, smaller interceptive errors were associated with more accurate tracking of the target trajectories and with a more stable oculomotor behavior prior to the interceptive response, supporting further the idea that efferent oculomotor information could contribute to the predictive estimates of the target motion necessary for the interceptive action.

## Putative neural substrates

**Figure 8:**
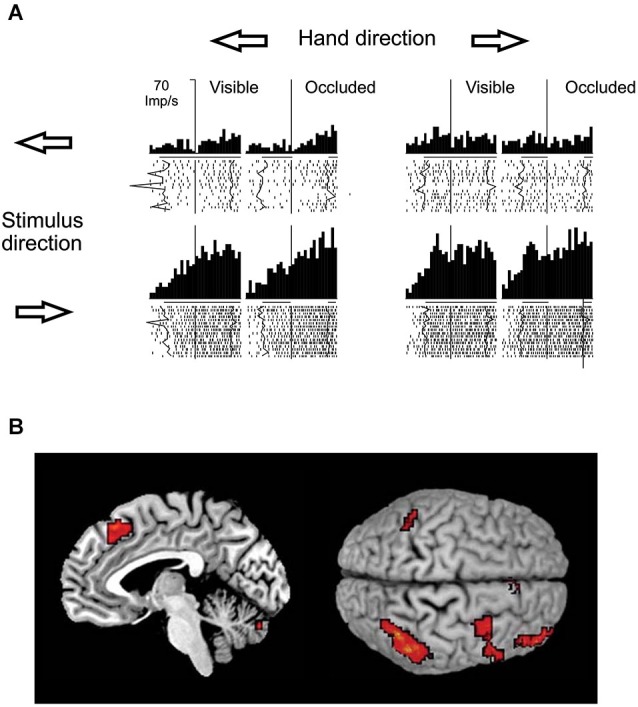
**Putative neural substrates of visual motion extrapolation**. **(A)** Responses of single neurons in monkey’s area LIP. The monkey used a joystick to guide a spot to a circular target, with vision of the moving spot prevented for the first 300–400 ms in occluded trials. Arrows indicate the directions of the hand movement and of the visual spot. The raster plots and histograms are aligned at the start of joystick movement, which corresponds to the visual spot disappearance on occluded trials (vertical line). Thick black lines beneath the histograms indicate the time during which the spot was visible, while line gaps on occluded trial histograms indicate the time during which the moving spot was not visible, on average. Jagged lines in raster plots follow spot onset and spot reappearance events on single trials. Note the persistence of the LIP neuron’s firing activity during periods of visual occlusion. Modified with permission from Eskandar and Assad (1999). **(B)** Mesial projection of the right hemisphere (left panel) and dorsal view (right panel, left hemisphere above) of the brain illustrate activity associated with temporal–spatial perceptual prediction. Statistical parametric map highlights voxels with significantly higher activity in the temporal–spatial task relative to the purely spatial task. Activation foci are located in the anterior inferior parietal cortex, in the ventral premotor cortex, in the dorsolateral prefrontal cortex, in the anterior pre-supplementary motor area and in the cerebellum. Modified with permission from O’Reilly et al. (2008).

With respect to the brain regions potentially involved in visual motion extrapolation processes, both monkey neurophysiology and human neuroimaging studies attribute a critical role to the posterior parietal cortex, particularly to the lateral intraparietal sulcus (LIP) in monkeys and to the posterior part of the human intraparietal sulcus (IPS), which is considered as the human homologue of the simian LIP. Strong evidence has come primarily from a series of electrophysiological studies in monkeys carried out by Assad and collaborators. By comparing the activity of parietal neurons during full vision, target motion occlusion and visual target blinks, it was found that parietal neurons retain a sustained activity during the occlusion condition that might represent inferred target motion in the absence of visual input or motor output (Assad and Maunsell, 1995). Eskandar and Assad (1999) investigated this issue further by comparing the activity of LIP, medial superior temporal area (MST) and medial intraparietal area (MIP) neurons while monkeys used a joystick to guide a spot on a computer display from the center of the screen to peripheral targets. In some trials, vision of the moving spot was occluded. They used information theory analysis to determine the type of information transmitted by the neurons in the three cortical areas, and found that LIP had the highest percentage of neurons whose activity represented inferred motion of the spot, while MST and MIP activity was best related to visual motion and hand motion, respectively (see Figure 8A). However, neurons in MST and MIP could also transmit information about inferred motion. This idea has received further support by neuroimaging and electroencephalographic studies in humans that have reported consistent activations of the posterior parietal cortex, particularly of the IPS, during oculomotor and manual tasks requiring visual extrapolation of occluded targets (Lencer et al., 2004; Olson et al., 2004; Ogawa and Inui, 2007; Shuwairi et al., 2007; O’Reilly et al., 2008; Beudel et al., 2009; Makin et al., 2009b).

In addition to the extrapolation of occluded targets, the role of posterior parietal cortex (area 7a) has been investigated with respect to the manual interception of constant speed, circular RM and LAM, and compared with that of the primary motor cortex in the monkey (Merchant et al., 2004a,b,c, 2005). Stimulus-related activity has been found to prevail in area 7a and hand-related activity in the motor cortex (see also Merchant et al., 2001). Moreover, neural activity was selectively associated with the stimulus angle during RM, whereas it was tightly correlated to the time-to-contact in the LAM condition, particularly in the motor cortex (see Merchant and Georgopoulos, 2006).

Premotor areas also appear to be involved consistently in visual extrapolation processes. For example, in studies where the experimental paradigm involved the production of an oculomotor response, such as a saccade to the moving target or a smooth pursuit movement, activity within the frontal eye-fields (FEF) during the periods of target occlusion showed activity compatible with this idea (Fukushima et al., 2002, 2008; Barborica and Ferrera, 2003, 2004; Xiao et al., 2007; Ferrera and Barborica, 2010). Similarly, human FEF, along with the prefrontal cortex, the angular gyrus and parieto-insular vestibular cortex (PIVC), seem to be engaged during maintenance of ocular tracking in the absence of a visual target (Nagel et al., 2006). Moreover, when tasks involve a manual response and/or temporal predictions, activations of human supplementary motor areas (SMA and pre-SMA) and, to a lesser extent, the premotor cortex, have been reported in relation to transient occlusions of visual targets (Ogawa and Inui, 2007; Shuwairi et al., 2007; O’Reilly et al., 2008; Beudel et al., 2009). Instead, the evidence that visual motion areas may also contribute to visual motion extrapolation processes is more controversial. For example, while Assad’s studies failed to show consistent activity related to target motion during visual occlusion in monkey area MST, Thier et al. have shown activity in the same area (but not in MT) related to the motion of “imaginary” targets (Ilg, 2003; Ilg and Thier, 2003). Also in human fMRI studies, activation of area hV5/MT+ during visual target occlusion has not been consistently found. However, the finding reported by Olson et al. (2004) that activity in hV5/MT+ was elevated during the occlusion period with a similar trend as that observed in the IPS may be suggestive of the possibility that hV5/MT+ does not exert a purely sensory function, but may also contribute to predictive processes. This could be consistent with other fMRI evidence that hV5/MT+ activity may be modulated by stimulus predictability and with results of transcranial magnetic stimulation studies showing that that hV5/MT+ may be involved in the processing of temporal predictions for target interception, as well as in motion prediction processes underlying apparent motion perception (Bosco et al., 2008; Alink et al., 2010; Vetter et al., 2013). In addition, visual motion extrapolation processes appear to involve also subcortical structures like the cerebellum, as suggested by neuroimaging studies that reported cerebellar or basal ganglia activations along with cortical areas (O’Reilly et al., 2008; Beudel et al., 2009). Direct electrophysiological evidence, in this respect, comes from Purkinje cells recordings in the D2 zone of the cat cerebellum, which showed that Purkinje cells maintain simple spike tonic activity during transient disappearance of a moving visual target, possibly reflecting the use of an internal model based on memory of the previous target motion (Cerminara et al., 2009).

Visual motion extrapolation in complex perceptual and motor tasks may require coordinated activity in many of these brain regions (see Figure 8B). In fact, functional connectivity analyses carried out by the study of O’Reilly et al. (2008) obtained good indication that temporal predictions resulting from visual extrapolation processes may be subtended by interplay between subcortical structures (such as, the cerebellum and the basal ganglia) with a parieto-frontal cortical network, including IPS, the FEF and V5/MT+.

**Figure 9:**
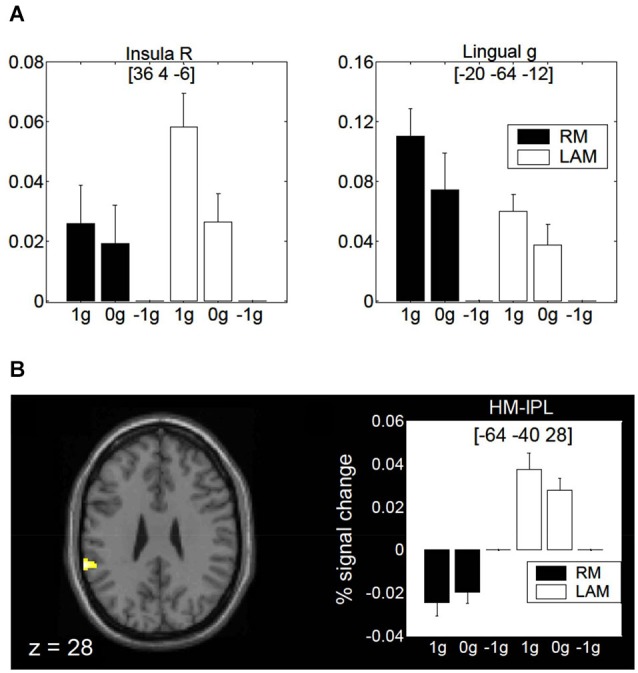
**Brain activity in response to gravitational motion and LAM**. **(A)** Mean activity profiles (±SEM) for the insula (left) and the lingual gyrus (right) foci showing statistically significant effect of gravitational motion. Black bars refer to activity recorded during RM trials, whereas white bars indicate activity levels for LAM trials. Signal changes are plotted as a function of the stimulus conditions and are expressed as percent change with respect to −1 g trials. **(B)** fMRI effects of 1 g motion specific to LAM. The left panel shows the statistical parametric map overlaid on an MNI template, indicating a significant focus of activation in a high-order motion region of the left inferior parietal lobe (HM-IPL). The right panel shows the mean activity profiles (±SEM) in the HM-IPL focus. BOLD signals are plotted as a function of the stimulus conditions and expressed as percent signal change relative to −1 g trials. Foci coordinates in panels **(A,B)** refer to the MNI standard space. Modified with permission from Maffei et al. (2010).

Finally, a specific role of the posterior insula and lingual gyrus for encoding gravity effects during interception of both linear RM and LAM has been revealed by fMRI (Maffei et al., 2010; Figure 9A). These regions seem to extract and process visual gravitational motion across a wide range of spatial and temporal frequencies (Maffei et al., 2010). In addition, a higher-order motion region located in the inferior parietal lobule close to the temporo-parietal junction (so called HM-IPL region) showed a preference for visual gravitational motion in LAM but not RM (Figure 9B). In previous studies, HM-IPL was described as a substrate for high-level motion processing based on salience, being engaged by color-salient, isoluminant gratings and by quartet-display LAM at 7 Hz (Claeys et al., 2003). These imaging findings fit with patients’ studies indicating that IPL lesions may impair LAM perception (Battelli et al., 2001).

In sum, visual motion extrapolation processes appear to be consistently reflected by activity in the IPS, with several other cortical and subcortical brain areas being engaged depending on the nature of the behavioral task (purely perceptual, oculomotor or manual interception) and of the visual motion stimulus (smooth or apparent motion).

## Conclusions

The present article reviewed experimental evidence, drawn mostly from the oculomotor and the manual interception literature, concerning the nature of visual motion extrapolation processes, which might allow efficient motor interactions with the environment when visual feedback is lacking or degraded. We concluded that several kinds of information contribute to visual motion extrapolation, including not only short term memory of the target kinematics, but also long-term information derived from heuristics, cognitive cues and internal representations of the physical properties of the visual environment. In particular, we discussed a growing body of evidence indicating that predictions of objects’ motion in a natural environment might be based on presupposed knowledge of gravity effects. Finally, with respect to the putative neural substrates, a critical role in maintaining a neural representation of the invisible target motion appears to be played by the intraparietal cortex together with other cortical and subcortical areas that may be engaged differentially depending on the nature of the behavioral task.

## Conflict of interest statement

The authors declare that the research was conducted in the absence of any commercial or financial relationships that could be construed as a potential conflict of interest.

## References

[B1] Adelson E. H. Bergen J. R. (1985). Spatiotemporal energy models for the perception of motion. J. Opt. Soc. Am. A 2, 284–299. 10.1364/josaa.2.000284 3973762

[B2] Alink A. Schwiedrzik C. M. Kohler A. Singer W. Muckli L. (2010). Stimulus predictability reduces responses in primary visual cortex. J. Neurosci. 30, 2960–2966. 10.1523/JNEUROSCI.3730-10.2010 20181593 PMC6633950

[B3] Assad J. A. Maunsell J. H. (1995). Neuronal correlates of inferred motion in primate posterior parietal cortex. Nature 373, 518–521. 10.1038/373518a0 7845463

[B4] Barborica A. Ferrera V. P. (2003). Estimating invisible target speed from neuronal activity in monkey frontal eye field. Nat. Neurosci. 6, 66–74. 10.1038/nn990 12483216

[B5] Barborica A. Ferrera V. P. (2004). Modification of saccades evoked by stimulation of frontal eye field during invisible target tracking. J. Neurosci. 24, 3260–3267. 10.1523/jneurosci.4702-03.2004 15056705 PMC6730017

[B6] Barnes G. R. Collins C. J. S. (2008). The influence of briefly presented randomized target motion on the extraretinal component of ocular pursuit. J. Neurophysiol. 99, 831–842. 10.1152/jn.01033.2007 18057108

[B7] Battaglia-Mayer A. Buiatti T. Caminiti R. Ferraina S. Lacquaniti F. Shallice T. (2014). Correction and suppression of reaching movements in the cerebral cortex: physiological and neuropsychological aspects. Neurosci. Biobehav. Rev. 42, 232–251. 10.1016/j.neubiorev.2014.03.002 24631852

[B8] Battelli L. Cavanagh P. Intriligator J. Tramo M. J. Hénaff M. A. Michèl F.. (2001). Unilateral right parietal damage leads to bilateral deficit for high-level motion. Neuron 32, 985–995. 10.1016/s0896-6273(01)00536-0 11754832

[B9] Becker W. Fuchs A. F. (1985). Prediction in the oculomotor system: smooth pursuit during transient disappearance of a visual target. Exp. Brain Res. 57, 562–575. 10.1007/bf00237843 3979498

[B10] Benguigui N. Bennett S. J. (2010). Ocular pursuit and the estimation of time-to-contact with accelerating objects in prediction motion are controlled independently based on first-order estimates. Exp. Brain Res. 202, 327–339. 10.1007/s00221-009-2139-0 20039024

[B11] Bennett S. J. Barnes G. R. (2003). Human ocular pursuit during the transient disappearance of a visual target. J. Neurophysiol. 90, 2504–2520. 10.1152/jn.01145.2002 14534275

[B12] Bennett S. J. Barnes G. R. (2004). Predictive smooth ocular pursuit during the transient disappearance of a visual target. J. Neurophysiol. 92, 578–590. 10.1152/jn.01188.2003 14960562

[B13] Bennett S. J. Barnes G. R. (2005). Timing the anticipatory recovery in smooth ocular pursuit during the transient disappearance of a visual target. Exp. Brain Res. 163, 198–203. 10.1007/s00221-004-2164-y 15821934

[B14] Bennett S. J. Barnes G. R. (2006a). Smooth ocular pursuit during the transient disappearance of an accelerating visual target: the role of reflexive and voluntary control. Exp. Brain Res. 175, 1–10. 10.1007/s00221-006-0533-4 16761137

[B15] Bennett S. J. Barnes G. R. (2006b). Combined smooth and saccadic ocular pursuit during the transient occlusion of a moving visual object. Exp. Brain Res. 168, 313–321. 10.1007/s00221-005-0101-3 16180042

[B16] Bennett S. J. Baures R. Hecht H. Benguigui N. (2010a). Eye movements influence estimation of time-to-contact in prediction motion. Exp. Brain Res. 206, 399–407. 10.1007/s00221-010-2416-y 20862463

[B17] Bennett S. J. Benguigui N. (2013). Is acceleration used for ocular pursuit and spatial estimation during prediction motion? PLoS One 8:e63382. 10.1371/journal.pone.0063382 23696822 PMC3656031

[B18] Bennett S. J. O’Donnell D. Hansen S. Barnes G. R. (2012). Facilitation of ocular pursuit during transient occlusion of externally-generated target motion by concurrent upper limb movement. J. Vis. 12:17. 10.1167/12.13.17 23262149

[B19] Bennett S. J. Orban de Xivry J.-J. Barnes G. R. Lefèvre P. (2007). Target acceleration can be extracted and represented within the predictive drive to ocular pursuit. J. Neurophysiol. 98, 1405–1414. 10.1152/jn.00132.2007 17553954

[B20] Bennett S. J. Orban de Xivry J. J. Lefèvre P. Barnes G. R. (2010b). Oculomotor prediction of accelerative target motion during occlusion: long-term and short-term effects. Exp. Brain Res. 204, 493–504. 10.1007/s00221-010-2313-4 20556369

[B21] Beudel M. Renken R. Leenders K. L. de Jong B. M. (2009). Cerebral representations of space and time. Neuroimage 44, 1032–1040. 10.1016/j.neuroimage.2008.09.028 18951984

[B22] Bosco G. Carrozzo M. Lacquaniti F. (2008). Contributions of the human temporoparietal junction and MT/V5+ to the timing of interception revealed by transcranial magnetic stimulation. J. Neurosci. 28, 12071–12084. 10.1523/JNEUROSCI.2869-08.2008 19005072 PMC6671632

[B23] Bosco G. Delle Monache S. Lacquaniti F. (2012). Catching what we can’t see: manual interception of occluded fly-ball trajectories. PLoS One 7:e49381. 10.1371/journal.pone.0049381 23166653 PMC3498163

[B24] Brenner E. Driesen B. Smeets J. B. J. (2014). Precise timing when hitting falling balls. Front. Hum. Neurosci. 8:342. 10.3389/fnhum.2014.00342 24904380 PMC4033095

[B25] Brouwer A. M. Brenner E. Smeets J. B. J. (2002). Hitting moving objects: is target speed used in guiding the hand? Exp. Brain Res. 143, 198–211. 10.1007/s00221-001-0980-x 11880896

[B26] Cavanagh P. (1992). Attention-based motion perception. Science 257, 1563–1565. 10.1126/science.1523411 1523411

[B27] Cerminara N. L. Apps R. Marple-Horvat D. E. (2009). An internal model of a moving visual target in the lateral cerebellum. J. Physiol. 587, 429–442. 10.1113/jphysiol.2008.163337 19047203 PMC2670054

[B28] Claeys K. Lindsey D. De Schutter E. Orban G. (2003). A higher order motion region in human inferior parietal lobule: evidence from fMRI. Neuron 40, 631–642. 10.1016/s0896-6273(03)00590-7 14642285

[B29] Collins C. J. S. Barnes G. R. (2006). The occluded onset pursuit paradigm: prolonging anticipatory smooth pursuit in the absence of visual feedback. Exp. Brain Res. 175, 11–20. 10.1007/s00221-006-0527-2 16724175

[B30] de Brouwer S. Missal M. Lefèvre P. (2001). Role of retinal slip in the prediction of target motion during smooth and saccadic pursuit. J. Neurophysiol. 86, 550–558. 11495930 10.1152/jn.2001.86.2.550

[B31] Delle Monache S. Lacquaniti F. Bosco G. (2015). Eye movements and manual interception of ballistic trajectories: effects of law of motion perturbations and occlusions. Exp. Brain Res. 233, 359–374. 10.1007/s00221-014-4120-9 25311389

[B32] DeLucia P. R. (2004). Time-to-contact judgments of an approaching object that is partially concealed by an occluder. J. Exp. Psychol. Hum. Percept. Perform. 30, 287–304. 10.1037/0096-1523.30.2.287 15053689

[B33] DeLucia P. R. Liddell G. W. (1998). Cognitive motion extrapolation and cognitive clocking in prediction motion task. J. Exp. Psychol. Hum. Percept. Perform. 24, 901–914. 10.1037//0096-1523.24.3.901 9627424

[B34] de Rugy A. Marinovic W. Wallis G. (2012). Neural prediction of complex accelerations for object interception. J. Neurophysiol. 107, 766–771. 10.1152/jn.00854.2011 22090456

[B35] De Sá Teixeira N. A. Hecht H. Oliveira A. M. (2013). The representational dynamics of remembered projectile locations. J. Exp. Psychol. Hum. Percept. Perform. 39, 1690–1699. 10.1037/a0031777 23398260

[B36] de’Sperati C. Deubel H. (2006). Mental extrapolation of motion modulates responsiveness to visual stimuli. Vision Res. 46, 2593–2601. 10.1016/j.visres.2005.12.019 16545854

[B37] Dessing J. C. Oostwoud Wijdenes L. Peper C. L. E. Beek P. J. (2009). Adaptations of lateral hand movements to early and late visual occlusion in catching. Exp. Brain Res. 192, 669–682. 10.1007/s00221-008-1588-1 18936928

[B38] Diaz G. Cooper J. Hayhoe M. (2013b). Memory and prediction in natural gaze control. Philos. Trans. R. Soc. Lond. B Biol. Sci. 368:20130064. 10.1098/rstb.2013.0064 24018726 PMC3758207

[B39] Diaz G. Cooper J. Rothkopf C. Hayhoe M. (2013a). Saccades to future ball location reveal memory-based prediction in a virtual-reality interception task. J. Vis. 13:20. 10.1167/13.1.20 23325347 PMC3587002

[B40] Dubrowski A. Lam J. Carnahan H. (2000). Target velocity effects on manual interception kinematics. Acta Psychol. (Amst) 104, 103–118. 10.1016/s0001-6918(99)00056-6 10769942

[B41] Eggert T. Rivas F. Straube A. (2005). Predictive strategies in interception tasks: differences between eye and hand movements. Exp. Brain Res. 160, 433–449. 10.1007/s00221-004-2028-5 15551090

[B42] Eskandar E. N. Assad J. A. (1999). Dissociation of visual, motor and predictive signals in parietal cortex during visual guidance. Nat. Neurosci. 2, 88–93. 10.1038/4594 10195185

[B43] Faisal A. A. Wolpert D. M. (2009). Near optimal combination of sensory and motor uncertainty in time during a naturalistic perception-action task. J. Neurophysiol. 101, 1901–1912. 10.1152/jn.90974.2008 19109455 PMC2695629

[B44] Ferrera V. P. Barborica A. (2010). Internally generated error signals in monkey frontal eye field during an inferred motion task. J. Neurosci. 30, 11612–11623. 10.1523/JNEUROSCI.2977-10.2010 20810882 PMC2942960

[B45] Fukushima K. Akao T. Shichinohe N. Nitta T. Kurkin S. Fukushima J. (2008). Predictive signals in the pursuit area of the monkey frontal eye fields. Prog. Brain Res. 171, 433–440. 10.1016/s0079-6123(08)00664-X 18718338

[B46] Fukushima K. Yamanobe T. Shinmei Y. Fukushima J. (2002). Predictive responses of periarcuate pursuit neurons to visual target motion. Exp. Brain Res. 145, 104–120. 10.1007/s00221-002-1088-7 12070750

[B47] Gregory R. L. Harris J. P. (1984). Real and apparent movement nulled. Nature 307, 729–730. 10.1038/307729a0 6700700

[B48] Hosking S. G. Crassini B. (2010). The effects of familiar size and object trajectories on time-to-contact judgements. Exp. Brain Res. 203, 541–552. 10.1007/s00221-010-2258-7 20440609

[B49] Huber S. Krist H. (2004). When is the ball going to hit the ground? Duration estimates, eye movements and mental imagery of object motion. J. Exp. Psychol. Hum. Percept. Perform. 30, 431–444. 10.1037/0096-1523.30.3.431 15161377

[B122] Ilg U. J. (2003). Visual-tracking neurons in area MST are activated during anticipatory pursuit eye movements. Neuroreport 14, 2219–2223. 10.1097/01.wnr.0000098750.87269.3e 14625451

[B123] Ilg U. J. Thier P. (2003). Visual tracking neurons in primate area MST are activated by smooth-pursuit eye movements of an “imaginary” target. J. Neurophysiol. 90, 1489–1502. 10.1152/jn.00272.2003 12736240

[B50] Kaas A. Weigelt S. Roebroeck A. Kohler A. Muckli L. (2010). Imagery of a moving object: the role of occipital cortex and human MT/V5. Neuroimage 49, 794–804. 10.1016/j.neuroimage.2009.07.055 19646536

[B51] Katsumata H. Russell D. M. (2012). Prospective versus predictive control in timing of hitting a falling ball. Exp. Brain Res. 216, 499–514. 10.1007/s00221-011-2954-y 22120106

[B52] Kattoulas E. Smyrnis N. Mantas A. Evdokimidis I. Raos V. Moschovakis A. (2008). Arm movement metrics influence saccade metrics when looking and pointing towards a memorized target location. Exp. Brain Res. 189, 323–338. 10.1007/s00221-008-1427-4 18512050

[B53] Kerzel D. (2003a). Attention maintains mental extrapolation of target position: irrelevant distractors eliminate forward displacement after implied motion. Cognition 88, 109–131. 10.1016/s0010-0277(03)00018-0 12711155

[B54] Kerzel D. (2003b). Mental extrapolation of target position is strongest with weak motion signals and motor responses. Vision Res. 43, 2623–2635. 10.1016/s0042-6989(03)00466-8 14552804

[B55] Kowler E. Martins A. J. Pavel M. (1984). The effect of expectations on slow oculomotor control-IV. Anticipatory smooth eye movements depend on prior target motions. Vision Res. 24, 197–210. 10.1016/0042-6989(84)90122-6 6719834

[B56] Kowler E. Steinman R. M. (1981). The effect of expectations on slow oculomotor control-III. Guessing unpredictable target displacements. Vision Res. 21, 191–203. 10.1016/0042-6989(81)90113-9 7269296

[B57] Kveraga K. Fendrich R. Hughes H. C. (2001). Ocular pursuit of predicted motion trajectories. Exp. Brain Res. 138, 393–397. 10.1007/s002210100701 11460778

[B58] Lacquaniti F. Bosco G. Indovina I. La Scaleia B. Maffei V. Moscatelli A.. (2013). Visual gravitational motion and the vestibular system in humans. Front. Integr. Neurosci. 7:101. 10.3389/fnint.2013.00101 24421761 PMC3872780

[B59] La Scaleia B. Lacquaniti F. Zago M. (2014). Neural extrapolation of motion for a ball rolling down an inclined plane. PLoS One 9:e99837. 10.1371/journal.pone.0099837 24940874 PMC4062474

[B60] Lee D. N. Reddish P. E. (1981). Plummeting gannets: a paradigm of ecological optics. Nature 293, 293–294. 10.1038/293293a0

[B61] Lencer R. Nagel M. Sprenger A. Zapf S. Erdmann C. Heide W.. (2004). Cortical mechanisms of smooth pursuit eye movements with target blanking. An fMRI study. Eur. J. Neurosci. 19, 1430–1436. 10.1111/j.1460-9568.2004.03229.x 15016102

[B62] López-Moliner J. Brenner E. Louw S. Smeets J. B. J. (2010). Catching a gently thrown ball. Exp. Brain Res. 206, 409–417. 10.1007/s00221-010-2421-1 20862460

[B63] Lu Z. L. Sperling G. (2001). Three-systems theory of human visual motion perception: review and update. J. Opt. Soc. Am. A Opt. Image Sci. Vis. 18, 2331–2370. 10.1364/josaa.18.002331 11551067

[B64] Lyon D. R. Waag W. L. (1995). Time course of visual extrapolation accuracy. Acta Psychol. (Amst) 89, 239–260. 10.1016/0001-6918(95)98945-z 7572268

[B65] Madelain L. Krauzlis R. J. (2003). Effects of learning on smooth pursuit during transient disappearance of a visual target. J. Neurophysiol. 90, 972–982. 10.1152/jn.00869.2002 12904499

[B66] Maffei V. Macaluso E. Indovina I. Orban G. Lacquaniti F. (2010). Processing of targets in smooth or apparent motion along the vertical in the human brain: an fMRI study. J. Neurophysiol. 103, 360–370. 10.1152/jn.00892.2009 19889846

[B67] Makin A. D. J. Poliakoff E. (2011). Do common systems control eye movements and motion extrapolation? Q. J. Exp. Psychol. (Hove) 64, 1327–1343. 10.1080/17470218.2010.548562 21480079

[B68] Makin A. D. J. Poliakoff E. Chen J. Stewart A. J. (2008). The effect of previously viewed velocities on motion extrapolation. Vision Res. 48, 1884–1893. 10.1016/j.visres.2008.05.023 18588909

[B69] Makin A. D. Poliakoff E. El-Deredy W. (2009b). Tracking visible and occluded targets: changes in event related potentials during motion extrapolation. Neuropsychologia 47, 1128–1137. 10.1016/j.neuropsychologia.2009.01.010 19350707

[B70] Makin A. D. Stewart A. J. Poliakoff E. (2009a). Typical object velocity influences motion extrapolation. Exp. Brain Res. 193, 137–142. 10.1007/s00221-008-1678-0 19139868

[B71] Marinovic W. Plooy A. M. Tresilian J. R. (2009). The utilisation of visual information in the control of rapid interceptive actions. Exp. Psychol. 56, 265–273. 10.1027/1618-3169.56.4.265 19439399

[B72] Marinovic W. Reid C. S. Plooy A. M. Riek S. Tresilian J. R. (2010). Delayed inhibition of an anticipatory action during motion extrapolation. Behav. Brain Funct. 6:22. 10.1186/1744-9081-6-22 20377911 PMC2859747

[B73] Marinovic W. Reid C. S. Plooy A. M. Riek S. Tresilian J. R. (2011). Corticospinal excitability during preparation for an anticipatory action is modulated by the availability of visual information. J. Neurophysiol. 105, 1122–1129. 10.1152/jn.00705.2010 21123661

[B74] Merchant H. Battaglia-Mayer A. Georgopoulos A. P. (2001). Effects of optic flow in motor cortex and area 7a. J. Neurophysiol. 86, 1937–1954. 11600652 10.1152/jn.2001.86.4.1937

[B75] Merchant H. Battaglia-Mayer A. Georgopoulos A. P. (2003). Interception of real and apparent motion targets: psychophysics in humans and monkeys. Exp. Brain Res. 152, 106–112. 10.1007/s00221-003-1514-5 12879173

[B76] Merchant H. Battaglia-Mayer A. Georgopoulos A. P. (2004a). Neural responses during interception of real and apparent circularly moving stimuli in motor cortex and area 7a. Cereb. Cortex 14, 314–331. 10.1093/cercor/bhg130 14754870

[B77] Merchant H. Battaglia-Mayer A. Georgopoulos A. P. (2004b). Neural responses in motor cortex and area 7a to real and apparent motion. Exp. Brain Res. 154, 291–307. 10.1007/s00221-003-1664-5 14579000

[B78] Merchant H. Battaglia-Mayer A. Georgopoulos A. P. (2004c). Neurophysiology of the parieto-frontal system during target interception. Neurol. Clin. Neurophysiol. 2004, 1–5. 16012629

[B79] Merchant H. Battaglia-Mayer A. Georgopoulos A. P. (2005). Decoding of path-guided apparent motion from neural ensembles in posterior parietal cortex. Exp. Brain Res. 161, 532–540. 10.1007/s00221-004-2100-1 15586277

[B80] Merchant H. Georgopoulos A. P. (2006). Neurophysiology of perceptual and motor aspects of interception. J. Neurophysiol. 95, 1–13. 10.1152/jn.00422.2005 16339504

[B81] Merchant H. Zarco W. Prado L. Pérez O. (2009). Behavioral and neurophysiological aspects of target interception. Adv. Exp. Med. Biol. 629, 201–220. 10.1007/978-0-387-77064-2_10 19227501

[B82] Mijatović A. La Scaleia B. Mercuri N. Lacquaniti F. Zago M. (2014). Familiar trajectories facilitate the interpretation of physical forces when intercepting a moving target. Exp. Brain Res. 232, 3803–3811. 10.1007/s00221-014-4050-6 25142150

[B83] Mitrani L. Dimitrov G. (1978). Pursuit eye movements of a disappearing moving target. Vision Res. 18, 537–539. 10.1016/0042-6989(78)90199-2 664335

[B84] Morris E. J. Lisberger S. G. (1987). Different responses to small visual errors during initiation and maintenance of smooth-pursuit eye movements in monkeys. J. Neurophysiol. 58, 1351–1369. 3437336 10.1152/jn.1987.58.6.1351

[B85] Mrotek L. A. Flanders M. Soechting J. F. (2004). Interception of targets using brief directional cues. Exp. Brain Res. 156, 94–103. 10.1007/s00221-003-1764-2 14722701

[B86] Mrotek L. A. Soechting J. F. (2007). Predicting curvilinear target motion through an occlusion. Exp. Brain Res. 178, 99–114. 10.1007/s00221-006-0717-y 17053910

[B87] Nagel M. Sprenger A. Zapf S. Erdmann C. Kömpf D. Heide W.. (2006). Parametric modulation of cortical activation during smooth pursuit with and without target blanking. an fMRI study. Neuroimage 29, 1319–1325. 10.1016/j.neuroimage.2005.08.050 16216531

[B121] Ogawa K. Inui T. (2007). Lateralization of the posterior parietal cortex for internal monitoring of self- versus externally generated movements. J. Cogn. Neurosci. 19, 1827–1835. 10.1162/jocn.2007.19.11.1827 17958485

[B88] Olson I. R. Gatenby J. C. Leung H. C. Skudlarski P. Gore J. C. (2004). Neuronal representation of occluded objects in the human brain. Neuropsychologia 42, 95–104. 10.1016/s0028-3932(03)00151-9 14615079

[B89] Orban de Xivry J.-J. Bennett S. J. Lefèvre P. Barnes G. R. (2006). Evidence for synergy between saccades and smooth pursuit during transient target disappearance. J. Neurophysiol. 95, 418–427. 10.1152/jn.00596.2005 16162830

[B90] Orban de Xivry J.-J. Missal M. Lefèvre P. (2008). A dynamic representation of target motion drives predictive smooth pursuit during target blanking. J. Vis. 8:6. 10.1167/8.15.6 19146290

[B91] O’Reilly J. X. Mesulam M. M. Nobre A. C. (2008). The cerebellum predicts the timing of perceptual events. J. Neurosci. 28, 2252–2260. 10.1523/JNEUROSCI.2742-07.2008 18305258 PMC6671847

[B92] Pola J. Wyatt H. J. (1997). Offset dynamics of human smooth pursuit eye movements: effects of target presence and subject attention. Vision Res. 37, 2579–2595. 10.1016/s0042-6989(97)00058-8 9373690

[B93] Port N. L. Pellizzer G. Georgopoulos A. P. (1996). Intercepting real and path-guided apparent motion targets. Exp. Brain Res. 110, 298–307. 10.1007/bf00228560 8836693

[B94] Regan D. Gray R. (2000). Visually guided collision avoidance and collision achievement. Trends Cogn. Sci. 4, 99–107. 10.1016/s1364-6613(99)01442-4 10689344

[B95] Rosander K. von Hofsten C. (2000). Visual-vestibular interaction in early infancy. Exp. Brain Res. 133, 321–333. 10.1007/s002210000413 10958522

[B96] Schiff W. Oldak R. (1990). Accuracy of judging time to arrival: effects of modality, trajectory and gender. J. Exp. Psychol. Hum. Percept. Perform. 16, 303–316. 10.1037//0096-1523.16.2.303 2142201

[B97] Sharp R. H. Whiting H. T. (1974). Exposure and occluded duration effects in a ball-catching skill. J. Mot. Behav. 6, 139–147. 10.1080/00222895.1974.10734990 23952726

[B98] Shuwairi S. M. Curtis C. E. Johnson S. P. (2007). Neural substrates of dynamic object occlusion. J. Cogn. Neurosci. 19, 1275–1285. 10.1162/jocn.2007.19.8.1275 17651002 PMC3133772

[B99] Soechting J. F. Flanders M. (2008). Extrapolation of visual motion for manual interception. J. Neurophysiol. 99, 2956–2967. 10.1152/jn.90308.2008 18436629

[B100] Soechting J. F. Juveli J. Z. Rao H. M. (2009). Models for the extrapolation of target motion for manual interception. J. Neurophysiol. 102, 1491–1502. 10.1152/jn.00398.2009 19571194 PMC2746781

[B101] Sokolov A. N. Ehrenstein W. H. Pavlova M. A. Cavonius C. R. (1997). Motion extrapolation and velocity transposition. Perception 26, 875–889. 10.1068/p260875 9509140

[B102] Sokolov A. Pavlova M. (2003). Timing accuracy in motion extrapolation: reverse effects of target size and visible extent of motion at low and high speeds. Perception 32, 699–706. 10.1068/p3397 12892430

[B103] Souto D. Kerzel D. (2013). Like a rolling stone: naturalistic visual kinematics facilitate tracking eye movements. J. Vis. 13:9. 10.1167/13.2.9 23390323

[B104] Spering M. Schütz A. C. Braun D. I. Gegenfurtner K. R. (2011). Keep your eyes on the ball: smooth pursuit eye movements enhance prediction of visual motion. J. Neurophysiol. 105, 1756–1767. 10.1152/jn.00344.2010 21289135

[B105] Tabata H. Miura K. Kawano K. (2008). Trial-by-trial updating of the gain in preparation for smooth pursuit eye movement based on past experience in humans. J. Neurophysiol. 99, 747–758. 10.1152/jn.00714.2007 18077667

[B106] Teixeira L. A. Chua R. Nagelkerke P. Franks I. M. (2006). Use of visual information in the correction of interceptive actions. Exp. Brain Res. 175, 758–763. 10.1007/s00221-006-0740-z 17051375

[B107] Tresilian J. R. (1995). Perceptual and cognitive processes in time-to-contact estimation: analysis of prediction-motion and relative judgment tasks. Percept. Psychophys. 57, 231–245. 10.3758/bf03206510 7885822

[B108] Vetter P. Grosbras M.-H. Muckli L. (2013). TMS over V5 disrupts motion prediction. Cereb. Cortex [Epub ahead of print]. 10.1093/cercor/bht297 24152544 PMC4380002

[B109] Vishton P. M. Reardon K. M. Stevens J. A. (2010). Timing of anticipatory muscle tensing control: responses before and after expected impact. Exp. Brain Res. 202, 661–667. 10.1007/s00221-010-2172-z 20135099

[B110] von Hofsten C. Kochukhova O. Rosander K. (2007). Predictive tracking over occlusions by 4-month-old infants. Dev. Sci. 10, 625–640. 10.1111/j.1467-7687.2007.00604.x 17683347

[B111] Watamaniuk S. N. J. Heinen S. J. (2003). Perceptual and oculomotor evidence of limitations on processing accelerating motion. J. Vis. 3, 698–709. 10.1167/3.11.5 14765954

[B112] Watson A. B. Ahumada A. J. (1985). Model of human visual-motion sensing. J. Opt. Soc. Am. A 2, 322–341. 10.1364/josaa.2.000322 3973764

[B113] Werkhoven P. Snippe H. P. Toet A. (1992). Visual processing of optic acceleration. Vision Res. 32, 2313–2329. 10.1016/0042-6989(92)90095-z 1288008

[B114] Wexler M. Klam F. (2001). Movement prediction and movement production. J. Exp. Psychol. Hum. Percept. Perform. 27, 48–64. 10.1037//0096-1523.27.1.48 11248940

[B115] Whiting B. Gill E. Stephenson J. (1970). Critical time intervals for taking in flight information in a ball-catching task. Ergonomics 13, 265–272. 10.1080/00140137008931141

[B116] Xiao Q. Barborica A. Ferrera V. P. (2007). Modulation of visual responses in macaque frontal eye field during covert tracking of invisible targets. Cereb. Cortex 17, 918–928. 10.1093/cercor/bhl002 16723405

[B117] Zago M. Iosa M. Maffei V. Lacquaniti F. (2010). Extrapolation of vertical target motion through a brief visual occlusion. Exp. Brain Res. 201, 365–384. 10.1007/s00221-009-2041-9 19882150

[B118] Zago M. Lacquaniti F. (2005). Visual perception and interception of falling objects: a review of evidence for an internal model of gravity. J. Neural Eng. 2, S198–S208. 10.1088/1741-2560/2/3/s04 16135884

[B119] Zago M. McIntyre J. Senot P. Lacquaniti F. (2008). Internal models and prediction of visual gravitational motion. Vision Res. 48, 1532–1538. 10.1016/j.visres.2008.04.005 18499213

[B120] Zago M. McIntyre J. Senot P. Lacquaniti F. (2009). Visuo-motor coordination and internal models for object interception. Exp. Brain Res. 192, 571–604. 10.1007/s00221-008-1691-3 19139857

